# Linking Online Gaming and Addictive Behavior: Converging Evidence for a General Reward Deficiency in Frequent Online Gamers

**DOI:** 10.3389/fnbeh.2014.00385

**Published:** 2014-11-10

**Authors:** Tim Hahn, Karolien Hilde Notebaert, Thomas Dresler, Linda Kowarsch, Andreas Reif, Andreas J. Fallgatter

**Affiliations:** ^1^Department of Psychiatry, Psychosomatics and Psychotherapy, University of Würzburg, Würzburg, Germany; ^2^Research Center of Marketing and Consumer Science, Katholieke Universiteit Leuven, Leuven, Belgium; ^3^Department of Psychiatry and Psychotherapy, University of Tübingen, Tübingen, Germany; ^4^LEAD Graduate School, University of Tübingen, Tübingen, Germany; ^5^Center of Integrative Neuroscience, Excellence Cluster, University of Tübingen, Tübingen, Germany

**Keywords:** massively multiplayer online role playing games, world of warcraft, resting-state fMRI, monetary incentive delay task, reward deficiency syndrome, behavioral activation system

## Abstract

Millions of people regularly play so-called massively multiplayer online role playing games (MMORPGs). Recently, it has been argued that MMORPG overuse is becoming a significant health problem worldwide. Symptoms such as tolerance, withdrawal, and craving have been described. Based on behavioral, resting state, and task-related neuroimaging data, we test whether frequent players of the MMORPG “World of Warcraft” (WoW) – similar to drug addicts and individuals with an increased risk for addictions – show a generally deficient reward system. In frequent players of the MMORPG “World of Warcraft” (WoW-players) and in a control group of non-gamers we assessed (1) trait sensitivity to reward (SR), (2) BOLD responses during monetary reward processing in the ventral striatum, and (3) ventral-striatal resting-state dynamics. We found a decreased neural activation in the ventral striatum during the anticipation of both small and large monetary rewards. Additionally, we show generally altered neurodynamics in this region independent of any specific task for WoW players (resting state). On the behavioral level, we found differences in trait SR, suggesting that the reward processing deficiencies found in this study are not a consequence of gaming, but predisposed to it. These findings empirically support a direct link between frequent online gaming and the broad field of behavioral and drug addiction research, thus opening new avenues for clinical interventions in addicted gamers and potentially improving the assessment of addiction-risk in the vast population of frequent gamers.

## Introduction

In so-called massively multiplayer online role playing games (MMORPGs), large numbers of users interact via the Internet. This genre – including games such as World of Warcraft (WoW), Lineage or EverQuest – has developed into a multi-billion dollar global market (DFC-Intelligence, 2004). In 2008, more than 16 million gamers participated in at least one such online game regularly (Woodcock, 2008). While gamers generally enjoy playing, overuse is widespread and well documented (Hsu et al., 2009). For instance, 45% of MMORPG players in the United States spend more than 20 h per week playing (Ng and Wiemer-Hastings, 2005). A governmental survey in South Korea indicated that 2.4% of young people are excessive game users (Faiola, 2006). Recently, it has been argued that MMORPG overuse is becoming a significant health problem worldwide (Hsu et al., 2009). This might be particularly true for MMORPGs such as WoW with their complex reward structure designed for maximum customer retention (Debeauvais et al., 2011).

Generally, signs of excessive gaming parallel symptoms of substance abuse and behavioral addictions. Symptoms include tolerance, withdrawal, craving, and negative life consequences (Young, 1996; Charlton and Danforth, 2007). Against this background, it has been suggested that excessive online gaming could be considered a form of behavioral addiction (Griffiths, 2005). Indirectly supporting this view, neuroimaging studies have shown an involvement of the brain’s reward system in gaming similar to drug use (Koepp et al., 1998). In addition, frequent video gamers show higher gray matter volume and increased blood-oxygen-level-dependent (BOLD) activity in response to monetary losses in the ventral striatum than infrequent players (Kuhn et al., 2011), which is similar to those seen in addiction. Focusing specifically on subjects suffering from online game addiction, evidence furthermore suggests that the neural correlates of cue-induced gaming urge are highly similar to those of cue-induced craving in substance dependence, again involving reward-related areas such as the ventral striatum (Ko et al., 2009).

Crucial for multiple forms of addiction, the mesolimbic reward system has been shown to play an essential role in the development and maintenance of addiction (Robbins and Everitt, 1999; Volkow et al., 2002). In short, addicted individuals and those at risk have been proposed to have a generally deficient reward system and that drug use can be seen as an attempt to compensate for this deficit (Blum et al., 1996). Corroborating this view, cocaine addicts show decreased activation of the ventral striatum in response to rewards unrelated to the drug itself (Goldstein et al., 2007). The same alterations in ventral-striatal reward processing were found in adolescent smokers (Peters et al., 2011), pathological gamblers (Reuter et al., 2005), and alcoholics (Beck et al., 2009).

It has been argued that such deficiencies are not a consequence of addiction, but causal to it. In line with this, personality traits such as high novelty seeking and low harm avoidance predict early substance abuse (Cloninger et al., 1988; Wills et al., 1994). Likewise, sensitivity to reward (SR) (Gray, 1982; Corr, 2008) is increased in high-risk as compared to low-risk drinkers in a sample of healthy young adults. Also, SR was negatively associated with age of onset of regular alcohol use in the same sample (Lyvers et al., 2009). Hinting at the physiological basis of this putative vulnerability to addiction, even adolescents who had smoked less than 10 times in their lives also showed deficient ventral-striatal responsiveness to rewards (Peters et al., 2011).

Against this background, the fundamental question arises whether frequent online gamers – similar to drug addicts and individuals with an increased risk for addictions – also show a generally deficient reward system. Such a deviation in the processing of rewards could provide insight into the neural underpinnings of persistent social problems and addiction-like behavior frequently observed in excessive gamers. Importantly, it would provide a direct link between online gaming and the broad field of behavioral and drug addiction research.

In order to test the general (i.e., not game-related) functioning of the reward-system in frequent online gamers, we assessed (1) trait SR, (2) BOLD responses during monetary reward processing in the ventral striatum, and (3) ventral-striatal resting-state dynamics in frequent players of the MMORPG “World of Warcraft” (WoW-players) and in a control group of non-gamers. In accordance with the Reward Deficiency approach, we hypothesized decreased responsiveness of the ventral striatum to rewards unrelated to the game itself. Providing further evidence for functional deviations in the mesolimbic reward system – independent of external stimuli – we secondly expected differences in local resting-state synchronization in WoW players as compared to non-gamers in the ventral striatum. Indicating a potentially increased vulnerability for addictions, we furthermore expected increased trait SR in WoW players as compared to non-gamers.

## Materials and Methods

### Participants

Sixteen male subjects who reported to frequently play the MMORPG “WoW” were recruited from the local community by advertisements, e.g., specialized computer game shops. Frequent online gaming was defined as playing at least four times per week for 1 h or more for at least 1 year. This relatively low threshold in conjunction with the exclusion of participants, who reported to have a known psychiatric disorder (see below), ensures the inclusion of frequent gamers who are potentially at risk for addiction without confounding results with addiction-related or other psychiatric disorders. Three subjects had to be excluded from further analyses due to technical error (1), psychiatric disorders (1), and/or excessive head motion (>3 mm/degree in any direction). Thus, 13 WoW players between 18 and 34 years of age (mean = 25.5; SD = 4.18) participated in this study; i.e., a resting-state session and a monetary incentive delay (MID) task. Data from 17 healthy male control subjects who reported not to play computer or video-games at all were also acquired. From the control sample, three (four) subjects had to be excluded from further analyses due to technical error (2) and/or excessive head motion (>3 mm/degree in any direction; one in the reward paradigm; two resting-state datasets). Thus, 14 controls between 21 and 30 years of age (mean = 24.5; SD = 2.85) entered resting-state analyses while 15 controls (mean age = 24.7; SD = 2.87) entered MID-task analyses. None reported to take illicit drugs or have a known psychiatric disorder according to self-report based on the German version of the Structured Clinical Interview for DSM-IV (SCID-I; Wittchen et al., 1997) Screening Questionnaire. After excluding some of the subjects for the above-mentioned reasons the resulting groups did not differ in age (*p* > 0.375) or smoking status (*p* > 0.154). All but one player and one control subject were right-handed, had normal or corrected-to-normal vision and reported to currently not take any medication. Written informed consent was obtained after detailed explanation of the study protocol. The study was approved by the Ethics Committee of the University of Wuerzburg, and all procedures involved were in accordance with the sixth revision of the Declaration of Helsinki.

### Psychometric testing

All participants completed the Sensitivity to Punishment (SP) and Sensitivity to Reward Questionnaire (SPSRQ; Torrubia et al., 2001) in its German version (Hewig and Hagemann; Der SPSR-Fragebogen von Torrubia, Avila, Molto, und Caseras; unpublished German translation, University of Trier; personal communication). The SPSRQ is a 48-item self-report measure of Gray’s trait anxiety and impulsivity dimensions. It consists of two scales, representing SR and SP. They are particularly designed to measure Gray’s concepts by linking SR to the behavioral activation system (BAS) and SP to the behavioral inhibition system (BIS). The two scales show retest reliabilities of 0.87 and 0.89 for the reward and the punishment scale, respectively, and good construct validity has also been shown (Sava and Sperneac, 2006).

### Reward task

We conducted a modified version of the MID task developed by Knutson et al. (2001) and used previously by our group (Hahn et al., 2009, 2011) which consisted of 60 trials, each of 10 s duration. In each of the trials, participants could win a certain amount of money when they pressed a button within a particular timeframe. During each trial, participants saw one of three different cue shapes (presentation time 2000 ms each) followed by a fixation cross as they waited a variable interval (2250 – 2750 ms). Each cue signaled the possibility of winning 0.05€ (*n* = 20; a circle with one horizontal line, i.e., a small reward) or 1.00€ (*n* = 20; a circle with three horizontal lines, i.e., a large reward). The third cue (*n* = 20; a triangle) indicated that no money could be won during this trial (i.e., control trial). Thereafter, they responded with a button press to a white target square which appeared for a variable length of time depending on the subject’s performance. Specifically, the mean reaction time obtained from the 10 practice trials was used as the initial target duration. It was increased by 30 ms if the subject failed to respond fast enough on more than one out of the last three consecutive trials. Likewise, it was decreased by 30 ms if the subject succeeded on more than two out of the last three consecutive trials. With this, we sought to ensure participants’ success on an average of 66% of the trials, thereby yielding a proportion of hits and misses comparable to that reported by Knutson et al. (2001). Additionally, target duration was set as to never decrease below 100 ms and never exceed 1000 ms. Feedback (2000 ms), which followed the disappearance of the target, informed participants of whether they had reacted in time during that trial and indicated their cumulative total win in Euros at that point. The three trial types (small reward, large reward, and control trial) were randomly ordered within the experiment.

### fMRI acquisition

Imaging was performed using a 1.5 T Siemens Magnetom Avanto TIM-system MRI scanner (Siemens, Erlangen, Germany) equipped with a standard 12-channel head coil. First, all subjects were scanned for 5 min. No specific instructions were given except to relax and hold still. In this session (henceforth called “resting-state data”), 11 10-mm-thick, interleaved axial slices (in-plane resolution: 4 mm × 4 mm) oriented at the AC–PC transverse plane were acquired with no interslice gap, using a T2*-sensitive single-shot EPI sequence with following parameters: repetition time (TR; 1480 ms), echo time (TE; 60 ms), flip angle (90°), matrix (64 × 64), field of view (FOV; 256 mm × 256 mm), and number of volumes (202).

Then, during the reward task, 24 4-mm-thick, interleaved axial slices (in-plane resolution: 3.28 mm × 3.28 mm) oriented at the AC-PC transverse plane were acquired with 1 mm interslice gap, using a T2*-sensitive single-shot EPI sequence with following parameters: repetition time (TR; 2000 ms), echo time (TE; 40 ms), flip angle (90°), matrix (64 × 64), field of view (FOV; 210 mm × 210 mm), and number of volumes (310). Stimuli were presented via MRI-compatible goggles (VisuaStim; Magnetic Resonance Technologies, Northridge, CA) using Presentation (Neurobehavioral Systems; http://www.neurobs.com). The first six volumes from the resting state as well as the task time-series were discarded to account for magnetization saturation effects.

### fMRI resting-state analysis

Resting-state analyses were conducted using the REST toolbox (Song et al., 2011) with the data processing assistant for resting-state fMRI (DPARSF; V2.1). First, resting-state data were slice-time corrected and head-motion correction (realignment) was applied. After that, the fMRI images were normalized to the Montreal Neurological Institute (MNI) template with a re-sampling voxel volume of 3 mm × 3 mm × 3 mm. After preprocessing, the time series for each voxel was detrended and bandpass-filtered (0.01–0.08 Hz) to reduce low-frequency drift and physiological high frequency respiratory and cardiac noise. Individual regional homogeneity (ReHo) maps were generated by calculating the Kendall’s coefficient concordance of the time series of a given voxel with those of its nearest neighbors (26 voxels) in a voxel-wise analysis. The resulting images were Fisher *z*-transformed and maps were spatially smoothed with a Gaussian kernel of 6 mm FWHM. SPM5 software and custom Matlab scripts were used to perform further second-level statistical testing (see below).

### Reward-task fMRI analysis

Task-related data were pre-processed and analyzed using Statistical Parametric Mapping software (SPM5, Wellcome Department of Cognitive Neurology, UK, implemented in Matlab 2011a, Math Works, Natick, MA, USA). Slice-time correction was applied and images were realigned. The mean image of the scans was computed and used as the source image for spatial normalization of the data. Data were then spatially smoothed, using an 8 mm FWHM Gaussian isotropic kernel. The time series in each voxel were high pass-filtered to 1/128 Hz to remove low-frequency noise and corrected for temporal auto-correlation using an autoregressive model with a lag of 1.

Contrast images for (a) 0.05€ reward anticipation vs. neutral condition (“small reward anticipation”) and (b) 1€ reward anticipation vs. neutral condition (“large reward anticipation”) then entered second-level analyses.

### Second-level analyses

All analyses were conducted within the ventral striatum as defined by the voxel mask from a publication-based probabilistic MNI atlas (http://hendrix.imm.dtu.dk/services/jerne/ninf/voi.html) at a probability threshold of 0.9 (Fox and Lancaster, 2002).

For reward-task data, a two-sample *t*-test was used to compare beta estimates in WoW players and non-gamers in a voxel-wise fashion yielding a *t*-value for each voxel in the ventral striatum. In order to assess significance of the results accounting for multiple comparisons, a permutation test based on cluster-sizes was conducted in the ventral striatum as follows: First, we computed *t*-maps comparing beta estimates for large (small) reward anticipation between WoW players and non-gamers, for each voxel and thresholded the map at a single voxel *p*-value of 0.01. Then, we repeated this procedure permuting group membership 100,000 times. For each of the permutation runs, we assessed the size of the largest voxel cluster obtained at the same single voxel *p*-value. Clusters showing a difference in beta estimate for large (small) reward anticipation between WoW players and non-gamers (single voxel *p*-value <0.01) were considered significant if a cluster was larger than 95% of the cluster-sizes obtained under permutation. This ensures a corrected α-level of 5% on the cluster level (for a highly related procedure, see Nichols and Holmes, 2002).

For lateralized analyses, a two-sample *t*-test was used to compare mean beta estimates in WoW players and non-gamers over the entire left and right ventral striatum, respectively. Resting-state ReHo differences between WoW players and non-gamers were computed in the same manner. Note that in accordance with our hypotheses, significance of *t*-tests relating to the reward-task data were one-tailed (expecting stronger reward-related responses in non-gamers than in WoW-players), while ReHo analyses were two-tailed.

## Results

**Figure 1:**
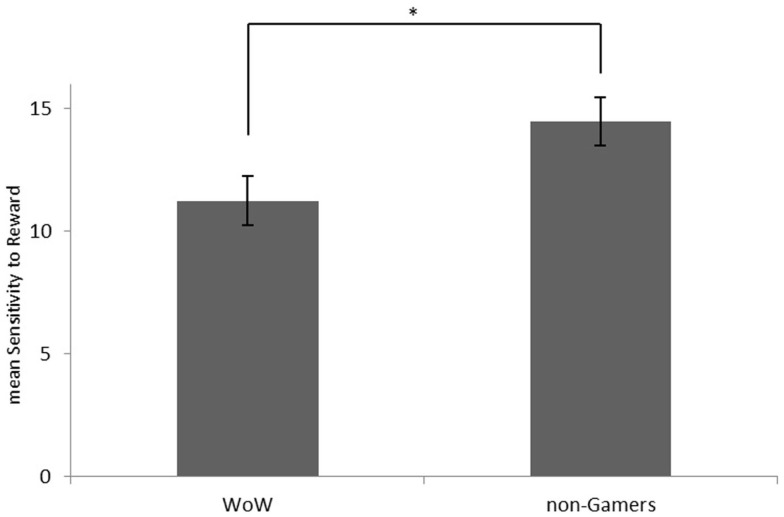
**Mean sensitivity to reward scores for WoW players and non-gamers**. *p < 0.05.

We found a difference in trait SR between gamers and non-gamers [*t*(26) = 2.30; *p* = 0.030, two-tailed; Figure 1]. Contrary to our expectations, however, WoW players showed significantly lower SR scores than non-gamers. We found no difference between the groups regarding trait SP [*t*(26) = 0.171; *p* = 0.866, two-tailed].

**Figure 2:**
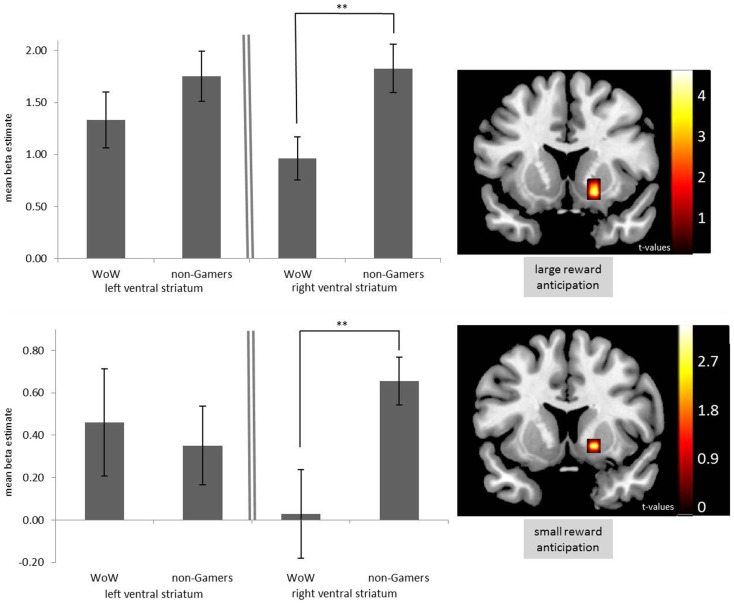
**Activation in the left and right ventral striatum for WoW players and non-gamers**. Top left panel shows response during the anticipation of large rewards. Bottom left panel shows activation during the anticipation of small rewards. Images display *t*-scores for the difference between WoW players and non-gamers (*p* < 0.05, corrected) for large (top right panel) and small reward condition (bottom right panel). **p < 0.01.

As hypothesized, WoW players showed significantly less activation in the ventral striatum during the anticipation of both small (*p* < 0.05, one-tailed, corrected; Figure 2, bottom right panel) and large rewards (*p* < 0.05, one-tailed, corrected; Figure 2, top right panel) compared to non-gamers. Investigating the mean responses during the anticipation of large rewards in the ventral striatum revealed that the difference between WoW players and non-gamers is significant in the right hemisphere only [right: *t*(26) = 2.74; *p* = 0.005, one-tailed; left: *t*(26) = 1.16; *p* = 0.128, one-tailed; Figure 2, top left panel]. Ventral-striatal responses to the anticipation of small rewards mirrored this effect [right: *t*(26) = 2.74; *p* = 0.005, one-tailed; left: *t*(26) = −0.36; *p* = 0.362, one-tailed; Figure 2, bottom left panel] albeit with markedly decreased activation over all groups as would be expected for the lower reward condition.

**Figure 3:**
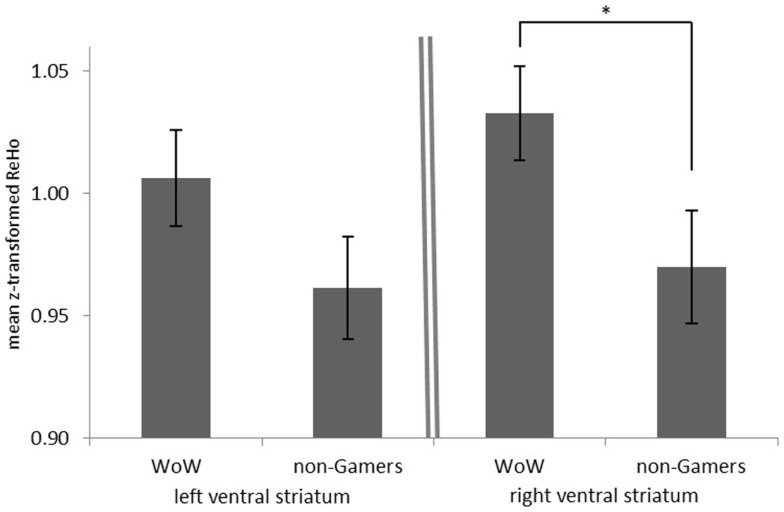
**Mean Fisher *z*-transformed regional homogeneity (ReHo) in the left and right ventral striatum for WoW players and non-gamers during resting state**. *p < 0.05.

Investigating resting-state synchronization in the same region, we observed an analogous pattern of results: ReHo in the right ventral striatum was increased in WoW players as compared to non-gamers [right: *t*(25) = −2.08; *p* = 0.048, two-tailed] while no such effect could be found in the left hemisphere [left: *t*(25) = −1.55; *p* = 0.135, two-tailed; Figure 3].

## Discussion

Mounting evidence suggests that addicted individuals have a general deficient reward system as suggested by Blum et al. (1996). Recent studies support this view for cocaine addicts (Goldstein et al., 2007), adolescent smokers (Peters et al., 2011), pathological gamblers (Reuter et al., 2005), and alcoholics (Beck et al., 2009). Here, we provide converging evidence for a general reward deficiency in frequent online gamers. Individuals who frequently play the MMORPG “WoW” displayed significantly decreased neural activation during the anticipation of both small and large monetary rewards in the ventral striatum. Strikingly, they showed a response to large rewards in the right ventral striatum that is in the range of the non-gamers’ response to small rewards whereas anticipation of small rewards did not elicit significant activation in WoW players at all. Importantly, the lower physiological reward responsiveness in WoW players shown independent of game-related stimuli implies a general deficiency which – given its occurrence when actual money is at stake – is likely to generalize to all those aspects of everyday life in which reward processing is essential. This deviation of neural responses might thus underlie persistent social problems and addiction-like behavior frequently observed in excessive gamers.

In addition, we provide evidence not just for dampened neural responses to monetary reward, but we show generally altered neurodynamics in the ventral striatum independent of any specific task by showing different local resting-state synchronization in WoW players as compared to non-gamers. While the exact physiological mechanisms causal to regional homogeneity (ReHo) remain unknown, it might be speculated that a habitually high synchronization of voxels in the striatum impairs its ability to respond to stimulation. While this would explain why higher ReHo was associated with decreased responsiveness to reward in our study, further investigations into the neural basis of ReHo are needed.

In line with the reward deficiency approach to addiction (Blum et al., 1996). Peters et al. (2011) showed that even adolescents who had smoked on 10 or less occasions in their lives showed dampened ventral-striatal responses during the anticipation of monetary rewards as compared to non-smokers. This evidence supports the notion that a generally deficient reward system as shown in the current study for frequent online gamers constitutes a vulnerability factor and not a consequence of gaming. Further corroborating this view, we found differences in trait SR between WoW players and non-gamers in this study (WoW players showed significantly lower SR scores than non-gamers). Given the definition of this trait as a stable behavioral tendency in response to reward across situations and the high retest reliability of this measure (Sava and Sperneac, 2006), it could be assumed that the reward processing deficiencies found in this study are not a consequence of gaming, but predisposed to it. Future research employing a longitudinal design will have to address this point in a more principled manner.

Two major limitations of our study have to be considered: First, the sample consists of male participants only. While the majority of WoW players (84%) is male (Yee, 2006), and our results thus ought to generalize to most gamers, further research might provide valuable insights into the neurophysiological differences in male and female gamers. Second, the sample size and the rather broad inclusion criteria for gamers call for a replication of our results in a larger sample with more detailed clinical information particularly regarding severity of addiction. On the other hand, combined evidence from multiple sources (personality, resting state, and task-related) in this study speaks to the validity of our findings. Furthermore, we investigated frequent WoW players, not people with a diagnosed addiction. Therefore, these subjects can only be regarded as potentially being at risk for addiction without showing signs of a psychiatric disorder. This prevents confounding factors from manifest addiction but may also limit the effects. In addition, online gamers – along with non-gaming Internet users – are likely to be younger, better educated, and earn more income than non-Internet users (Youn et al., 2003). In combination with other personal and social factors, this may prevent potentially harmful effects even in heavy users.

We also like to emphasize that the effects of video games on cognition and behavior are manifold and cannot be described as being exclusively positive or negative. There are findings that show the beneficial effects on personal well-being and cognitive function such as visual attention (Green and Bavelier, 2003). At the moment, however, experts agree there are still several issues and challenges that should be addressed to move the field forward (Bavelier et al., 2011).

In summary, we show converging evidence from behavioral as well as resting state and task-related neuroimaging data suggesting that frequent online gamers – similar to drug addicts and individuals with an increased risk for addictions – show a generally deficient reward system. Such a deviation in the processing of rewards helps to explain persistent social problems and addiction-like behavior frequently observed in excessive gamers. Crucially, it provides a direct link between frequent online gaming and the broad field of behavioral and drug addiction research. Thus, our findings might open new avenues for clinical interventions in addicted gamers and improve the assessment of addiction-risk in the vast population of frequent gamers.

## Conflict of Interest Statement

The authors declare that the research was conducted in the absence of any commercial or financial relationships that could be construed as a potential conflict of interest.
